# Developing Internet-Based Health Interventions: A Guide for Public Health Researchers and Practitioners

**DOI:** 10.2196/jmir.3770

**Published:** 2015-01-23

**Authors:** Keith J Horvath, Alexandra M Ecklund, Shanda L Hunt, Toben F Nelson, Traci L Toomey

**Affiliations:** ^1^Division of Epidemiology and Community HealthUniversity of MinnesotaMinneapolis, MNUnited States

**Keywords:** Internet, public health, intervention, development

## Abstract

**Background:**

Researchers and practitioners interested in developing online health interventions most often rely on Web-based and print resources to guide them through the process of online intervention development. Although useful for understanding many aspects of best practices for website development, missing from these resources are concrete examples of experiences in online intervention development for health apps from the perspective of those conducting online health interventions.

**Objective:**

This study aims to serve as a series of case studies in the development of online health interventions to provide insights for researchers and practitioners who are considering technology-based interventional or programmatic approaches.

**Methods:**

A convenience sample of six study coordinators and five principal investigators at a large, US-based land grant university were interviewed about the process of developing online interventions in the areas of alcohol policy, adolescent health, medication adherence, and human immunodeficiency virus prevention in transgender persons and in men who have sex with men. Participants were asked questions that broadly addressed each of the four phases of the User-Centered Design Process Map from the US Department of Health and Human Services' Research-Based Web Design & Usability Guidelines. Interviews were audio recorded and transcribed. Qualitative codes were developed using line-by-line open coding for all transcripts, and all transcripts were coded independently by at least 2 authors. Differences among coders were resolved with discussion.

**Results:**

We identified the following seven themes: (1) hire a strong (or at least the right) research team, (2) take time to plan before beginning the design process, (3) recognize that vendors and researchers have differing values, objectives, and language, (4) develop a detailed contract, (5) document all decisions and development activities, (6) use a content management system, and (7) allow extra time for testing and debugging your intervention. Each of these areas is discussed in detail, with supporting quotations from principal investigators and study coordinators.

**Conclusions:**

The values held by members of each participating organization involved in the development of the online intervention or program, as well as the objectives that are trying to be met with the website, must be considered. These defined values and objectives should prompt an open and explicit discussion about the scope of work, budget, and other needs from the perspectives of each organization. Because of the complexity of developing online interventions, researchers and practitioners should become familiar with the process and how it may differ from the development and implementation of in-person interventions or programs. To assist with this, the intervention team should consider expanding the team to include experts in computer science or learning technologies, as well as taking advantage of institutional resources that will be needed for successful completion of the project. Finally, we describe the tradeoff between funds available for online intervention or program development and the complexity of the project.

## Introduction

A goal of the US national initiative, Healthy People 2020, is to “Use health communication strategies and health information technology (IT) to improve population health outcomes and health care quality, and to achieve health equity [1]”. Health research plays an important role in reaching that goal by developing and testing Internet-based health promotion interventions. Internet interventions have been defined as “systematic treatment/prevention programs, usually addressing one or more determinants of health…delivered largely via the Internet…and interfacing with an end user” (p. 274) [2]. Internet interventions range from relatively simple computer-tailored messaging and education [3,4] to more sophisticated website interventions [5,6]. More recently, mobile interventions that use text messaging and social media apps have emerged [7-9].

Internet interventions have been developed across a broad range of health areas. Researchers in the areas of human immunodeficiency virus (HIV) prevention [7,8,10], smoking cessation [4,6,11], diabetes self-management [5,12], and nutrition/physical activity promotion [3,13,14] are among those experimenting with the benefits offered by the Internet. Although reviews of technology-assisted intervention studies are mixed [3,7,10,13], their success in modifying some health behaviors suggests that using technology to support health interventions is a promising approach [2].

Despite support for the feasibility and efficacy of Internet-based health interventions, relatively little guidance is available to researchers or practitioners who are interested in online interventions from colleagues with more experience in developing interventions that are wholly or partly delivered online. The process of online intervention development is complex and usually involves multiple teams of persons with different skills sets, objectives, and perspectives. In a review of computer-assisted instruction for medical education, it was noted that the inexperience of investigators in the use of technology for interventions may hinder advancement in the eHealth field [15]. Advice regarding how to develop effective websites is available from the computer science, marketing, and business professions (eg, [16-18]); however, these guidelines often take the perspective of how best to develop interventions that increase purchases or user traffic. Such advice may not be directly related to the goals and purposes of online health intervention research and practice.

Researchers and practitioners interested in developing online health interventions most often rely on Web-based [19] and print (see [20] for a list of print resources) resources to guide them through the process of online intervention development. Although useful for understanding many aspects of best practices for website development, missing from these resources are concrete examples of experiences in online intervention development for health apps from the perspective of those conducting online health interventions. To fill this gap, Bull [21] recently discussed theoretical and procedural aspects of developing, implementing, and evaluating technology-based health promotion interventions.

To provide greater context and guidance for those interested in developing technology-based health interventions, we conducted interviews with principal investigators and project coordinators who successfully developed Internet-based health interventions. The primary purpose of the interviews was to understand the challenges of such work and gain firsthand accounts of lessons learned from their experiences. We were particularly interested in investigators’ and coordinators’ experiences in working with vendors, defined here as an outside company that assisted with the overall design of the intervention and whose employees actually programmed the online health intervention. This study is meant as a series of case studies in the process of developing online health interventions to provide insights for researchers and practitioners who are considering similar technology-based interventional or programmatic approaches.

## Methods

### Participants

Persons associated with five unique research studies were interviewed for the purposes of this study. A convenience sample of six study coordinators (two from one research study) and five principal investigators at the University of Minnesota were interviewed about the process of developing online interventions in the areas of alcohol policy, adolescent health, medication adherence, and HIV prevention with men who have sex with men (MSM) and transgender persons. No incentives were provided to coordinators or principal investigators.

### Procedures

This study was not considered human subjects research since participants were not asked to provide information on themselves (rather they were asked to provide information about the research study); however, all research activities on the parent grant (under which this study was conducted) were approved by the University of Minnesota Institutional Review Board. One-on-one semistructured interviews with all but one respondent were conducted at their place of work, while one interview was conducted in a coffee house. Since the purpose of this study was to provide guidance to other researchers and practitioners in the process of online health intervention development, three of the authors (KJH, TT, and AE) who are involved in online health intervention research were interviewed by another study team member. Interviews were digitally recorded and lasted from 30-75 minutes. Audio recordings were transcribed in preparation for analysis.

### Interview Guide Development

Development of the interview guide was based on the Research-Based Web Design & Usability Guidelines from the US Department of Health and Human Services [19]. The guidelines address multiple facets of user experience-based website development, including the fundamentals of user experience, developing content for websites, project management, and best practices for developing a visually appealing website. The process of developing a user-centered website is presented graphically, and referred to as the User-Centered Design Process Map [22], in four separate but interrelated phases: (1) the Plan phase includes how the original idea was conceived, what team members were involved in initial planning, and hiring the vendor, (2) the Analyze phase encompasses the adaptation of an existing website or curriculum, learning about the target audience, and formative research, (3) the Design phase entails defining the individual components of the websites, discussions about authoring and uploading content, and work processes with the vendor, and (4) the Test and Refine phase describes processes for internal testing, usability testing, and de-bugging. For the purpose of this study, we asked participants questions that broadly addressed each of these four phases (eg, study staff and their roles, planning the intervention, formative research on the target population, online data collection, website design and testing, and issues of cost and timeline). Similar, but separate, interview guides were developed for principal investigators and study coordinators.

In addition to the semistructured interview, quantitative information about each study was extracted from the interview and shown in Table 1.

**Table 1:** Study, research team, and vendor characteristics.

		Study 1	Study 2	Study 3	Study 4	Study 5
**Study characteristics**						
	Topic	Alcohol Policy	Adolescent Health	MSM HIV Prevention	Transgender HIV Prevention	Medication Adherence
	Grant time length (years)	5	3	5	5	3
	Intervention development time (months)	18	12	24	30	13
	Study type	Efficacy trial	Pilot trial	Efficacy trial	Efficacy trial	Pilot trial
	Online only or hybrid (online & offline)	Hybrid	Online only	Online only	Online only	Online only
	Prior offline version	Yes	No	Yes	Yes	No
	Previous online version	No	No	Yes	Yes	No
	Development cost (US$)	235,000	35,000	300,000	300,000	45,000
**Research team**						
	PI: Experience with online research	No	No	Yes	Yes	Yes
	Coordinator: Experience with online research	No	No	No	No	Yes
**Vendor**						
	Prior vendor experience on research grant(s)	Yes	No	No	Yes	Yes
	Method vendor chosen	Peer referral	Peer referral	Prior project	Prior project	Peer referral
	Budget (at vs over vs under)	At budget	At budget	Over budget	Over budget	At budget
	Vendor employee size (Small: 1-10; Medium: 11-49; Large: 50+)	Medium	Small	Large	Medium	Small
	In close proximity of research team	Yes	Yes	Yes	Yes	No

### Data Analysis

The coding scheme was developed by 3 study authors (KH, SH, and AE), who collectively are referred to as the “coding team”. Qualitative codes were developed using line-by-line open coding for all transcripts [23], with the unit of analysis being a complete thought reflecting one of the codes. As such, the unit of analysis could range from several words to multiple sentences. The initial coding categories were determined by each member of the coding team independently coding two transcripts and convening to discuss mutually agreed upon and overlapping codes. For each category, a definition was agreed upon (the coding book and definitions are available from the first author upon request). Next, a second round of coding was conducted in which two transcripts were coded by 2 members of the coding team, after which the coding team met to finalize the coding scheme and definitions. Finally, all transcripts were coded by 2 members of the coding team. Participants who were also involved in the design and interpretation of this study did not code their own transcript to avoid potential coding bias. Each pair of coders met to discuss all of the statements assigned under each code, with disagreements being resolved through discussion [24]. Once all transcripts were coded, the results were presented in a debriefing session with all of the authors for input and interpretation. The themes presented below represent the culmination of this coding and debriefing process.

## Results

### Summary

An overview of the characteristics of each study is shown in Table 1, including general information about the study, the research team, and characteristics of the vendor that developed the intervention. Intervention studies were drawn from a range of topics. Studies with longer grant periods typically allocated more time for intervention development, had larger research budgets, and had products that were substantially more expensive. Three studies were preceded by an earlier offline or online version of the intervention. Three of the five studies had a principal investigator (PI) with prior experience with online research; however, only two of the project coordinators had such experience. Vendors, which ranged from those with few to many employees, were chosen either by a referral from a peer who had prior experience with the vendor, or because the vendor was used on an earlier version of the intervention. In all but one case, the vendor was within relatively close proximity to the research team, allowing for face-to-face meetings. Three of the projects met their budget estimate, while two of the projects exceeded their budget estimate. The projects that exceeded their budget estimate were both large efficacy trials with relatively large estimated budgets at the onset.

The themes below are organized by the phases of the User-Centered Design Process Map (ie, Plan, Analyze, Design, and Test and Refine), and generally are arranged in chronological order in which tasks should be considered or completed.

### Plan and Analyze

#### Overview

The themes identified under the Plan and the Analyze phases of the usability model include aspects of the study that prepare the team to develop the intervention, as well as conducting formative research to ensure that the intervention meets the needs of the target population.

#### Hire a Strong (or at Least the Right) Research Team

Assembling an intervention team that will ensure the success of the project is critical, as lacking expertise on the team or including team members who are not a good fit for technology-delivered projects may derail or postpone completion of the project. In addition to content experts who will be expected to develop and review content for the online intervention, hiring a study coordinator to act as a liaison between the intervention team and the vendor is essential to the success of the project. The coordinator will likely have the most day-to-day interaction with the vendor and must advocate on behalf of the intervention team. Coordinators devote much of their time to working closely with the vendor to ensure that the project remains on the timeline and within budget. Coordinators who had more experience with online research were aware of this responsibility and were able to more effectively communicate with the vendor regarding the budget and timeline:

I was pretty experienced in working in another intervention…It taught me a lot on how the academic process works with this. I’ve had a lot of experience with the vendors in technology projects, but I understand some of the academic process behind it and then being able to translate that to the vendor in a coherent manner…I think having someone who’s really good at project management and that technical translation piece is really important.

Coordinator, Medication Adherence

However, coordinators with no prior online intervention experience developed an understanding of the importance of their liaison role as they became more experienced in the position:

It was really challenging and in a lot of ways because I think we didn’t spell it out and there wasn’t anyone [to guide me through the process]. If I went through it again now I would know. As the study coordinator I would think that was my role to at least work with the vendor to see what their plan was or to make sure that there was a timeline laid out, but I didn’t know that at the time.

Coordinator, Adolescent Sexual Health

Interviewees reported that the coordinator must be available in order to respond to vendor requests during the development phase of the project, as well as be organized and detail oriented to track study progress (which is discussed in more detail under Design).

In addition to the coordinator, several persons noted that it is ideal to include team members who have experience in contract negotiations and someone who has expertise or experience in software programming. Contract negotiation is difficult without prior experience and may require team members to learn about contracts: “Certainly know what your contract is, know how to manage a contract, certainly understand what scope of work means and out of scope, and when is it legitimate to pay more money than what you agreed upon and when is it not legitimate to do so” (PI, Alcohol Policy). Including an expert in computer programming on the study team was critical since they could provide guidance to the study team about which programming language would be most sustainable for future iterations of the intervention and to advise the team about the appropriateness of the projected timeline and programming expense. The research team should assume that the online health intervention will go through multiple iterations in its life and that different persons and vendors may be working on the project over many years. Therefore, it is critical to develop the intervention using a common and widely used coding language since it may be necessary to hire different vendors to work on the code at different points in time.

#### Know Your Target Population and Anticipate How They Will Use Your Website

The importance of conducting formative research to understand their needs and preferences for intervention content and features has been well documented (eg, [25,26]). The same care should be taken to consider how target group members will react to and use different aspects of the technology. For example, it was noted by several persons that technology-based interventions are highly appropriate for some socially marginalized or isolated groups because members of these groups have been using technology to connect with one another:

The trans community are early adopters and are really interested in connecting online. Because they are such a marginalized community that has such a hard time connecting in person, that they were really quick and really eager to adopt online stuff…anything that’s come along basically the trans community has jumped right on board.

Coordinator, Transgender HIV Prevention

However, other investigators faced challenges of trying to develop an intervention that would appeal to both high and low computer literacy users:

Our target audience, which is mostly owners and managers of bars and restaurants, has an incredibly varied level of comfort with using the Internet. We have some managers who are…on the Internet every day for many hours a day…and are very computer savvy. We also work with a lot of owners and managers who might be older, who might not even have an email address, who are not nearly as comfortable with using the Internet. So it can really vary.

Coordinator, Alcohol Policy

Once the digital literacy of the target audience is established, anticipating how users will react to different website features can assist with guiding the development of the intervention. Wrongly anticipating how participants will interact with the technology was common and demonstrates the need for formative research and usability testing. For example, in a study of teen sexuality, the investigators released exercises for teens to complete on a regular basis; however, they soon realized that these activities were premature and users did not take advantage of the exercises until much later:

We would introduce a few each week to try to encourage teens to log on weekly, or a couple of times a week. What ended up happening is that they would all log on just at the very end of the month and try to get through all of the required tasks, even though we let them know that that was difficult and it was really time-consuming to do it that way.

PI, Adolescent Sexual Health

At least one researcher noted that the research team underestimated the sophistication of their target population, which hampered engagement in the online intervention. The potential mismatch between researchers’ expectations and actual use of the website by users should be anticipated to the degree possible prior to intervention launch and explored in usability or pilot testing to the extent possible.

#### Take Time to Plan Before Beginning the Design Process

Across researchers, it was apparent that much of the planning process could be accomplished before meeting with the vendor and writing the contract. Taking time to fully conceptualize the goals of the project and begin developing some of the content for the intervention will make the development process smoother once the vendor is engaged in the process. As persons with expertise in their field, researchers and practitioners will be required to develop content for the intervention. Some researchers noted that incorporating extra time in the grant timeline before hiring a vendor to conceptualize the project and begin some content development before the first meeting with the vendor would save time and money in the long run:

I think it would be very beneficial in the future to have a lot of your intervention created…before you get a Web vendor involved. Really think about who your target population is, what kind of Web experience they would want. Think about what you want your website to be able to do, particularly in the research environment where we want to collect all these outcome measures and process measures. Think about what kind of data you want to collect, what you want to get out of it.

Coordinator, Alcohol Policy

Developing content early will also help to avoid problems that could arise from simultaneously implementing the intervention while developing content for the intervention.

In sum, there are a number of critical steps and responsibilities that the intervention team needs to consider during the Plan and the Analyze phases of intervention development that may critically impact how smoothly the development process will go for the current intervention, as well as the long-term sustainability of the project.

### Design

#### Overview

The themes identified under the Design aspect of the usability model are those that relate to the intervention itself or the process of developing the intervention.

#### Vendors and Researchers Have Differing Values, Objectives, and Language

Across all persons interviewed for this study, the most prominent theme observed was that vendors and researchers often have different and sometimes competing values that they bring to the intervention development process. As a result, project staff and vendors often have different objectives when embarking upon the development of the intervention. Examples of values for researchers, based on our own experience, are shown in Table 2. These competing values and objectives may be due to the for-profit versus non-profit nature of each party, which can often lead to miscommunication between parties. For example, when asked about the most difficult part of the working relationship between the research team and the vendors, one participant stated: “Communication between the two agencies, different values. I mean at the end of the day they are a private company that needs to make a profit. At the end of the day we’re a public health agency that needs to have a deliverable that can go out” (PI, MSM HIV Prevention).

In addition, misunderstandings may arise because vendors are more familiar with working for for-profit organizations that need a website to interface with consumers of a product or service. The shift to working with academic institutions that value data privacy and proper data collection can create confusion:

They [the vendor] do a lot with [name of large international client] and not academic institutes and I think that was also the problem because they were more used to working on websites and doing stuff for companies…The user data is so valuable for us and they don’t think like researchers, so sometimes it was really hard for them to understand what we wanted.

Coordinator, MSM HIV Prevention

Part of the problems that arose during the working relationship came from the use of different language by members of the research team and vendors. For example, it may be clear to researchers what the term “intervention” means and what requirements are needed to conduct an intervention study, vendors who are not familiar with research may conceptualize an intervention closer to a commercial website. These values and language discrepancies were most often worked out by explicitly acknowledging the problem and understanding them as a necessary part of the learning experience.

Challenges between the research team and vendors were often described as “miscommunication” that could be worked out during the intervention development process, and additional time and explicit attention to these issues should be included into the overall timeline. However, there were instances where such miscommunication led to a more lasting mistrust between the two parties. Even though these competing values, objectives, and language were universal among researchers we talked with for this study, there were also agreed upon methods to manage such conflicts. These are discussed in the following two themes.

**Table 2:** Example values and objectives from a research perspective.

Task	Value(s)	Objective(s)	Action steps/Website features & functions
Overall	Advance science and understanding of complex behaviors and events	Test the efficacy of an online intervention to improve a health outcome	Develop a functional and effective online health intervention
Contract & Budget	Be a good steward of public/grant funds	Stay within budget; an effective intervention for the least cost	Choose a vendor with skills and processes to finish the intervention
		Advocate for the research team	Assign advocate to hold explicit discussions with the vendor about website needs and budget limitations
	Making sure that the intervention is theoretically driven, and sufficiently potent to change behavior	Conduct intervention development in a thoughtful and methodological manner	Provide extra time in the contract to make important intervention design decisions
Website Look & Feel	Create an intervention that is engaging to the target population	Develop an intervention with graphics, features and functions that the target population will use	Conduct formative research; allow time before development begins to understand the technological capabilities and needs of the target population
Data Collection, Management, & Security	Protect participant confidentiality	Develop strong security protocols	Discuss security needs with the vendor and the importance of security protocols.
			Develop security protocols to limit access to the intervention from outside sources
	Collect data to inform research and practice	Build databases that can hold participant and online intervention usage data	Include extra funds in contracts to develop “back-end” databases that can be easily accessed by the research team

#### Develop a Detailed Contract

One way to manage miscommunication and conflict between vendors and the intervention team was to develop a detailed contract that established the scope of work, the working relationship between the two sides, and agreement on the budget. The contract is a critical document throughout the entire development process. It provides the starting point for what features of the intervention will be built, how the intervention and vendor team will divide the work during the development process, and (perhaps most importantly) how development costs will be charged. The only research team interviewed as part of this study that had little difficulty with the contract was the one in which the project coordinator had extensive experience from prior projects in writing up detailed contracts. Most often, the inexperience of researchers to understand or know what level of detail to include in the contract resulted in problems during the development process: “And in the setting up the contract…not using clear enough language…it [the contract] should have been exact. We thought we were being exact and we realized we should be even more precise” (PI, Adolescent Sexual Health). In addition to helping the development process run smoothly as a result of clearly defined scope and roles, the contract was a document that was continually referred to throughout the development process to resolve conflict between the research team and vendor. Persons interviewed for this study frequently provided examples in which the research team and vendors disagreed on the scope of work or aspects of the budget. However, a detailed contract often provided a basis on which to resolve conflicts:

We had a couple of the online components where they gave us drafts that we just thought were completely off base in terms of the discussions we had been having. So we would send back and ask for multiple drafts and I think we didn’t get a lot of push back in some of the early stages but we ended up getting pushed back and they came back and said the contract only covered one round of reviews. We had to point out that the contract actually didn’t specify that. 

PI, Alcohol Policy

All persons interviewed agreed that it is ideal that the contract explicitly state that the negotiated cost of the subcontract be for the complete, final working version of the intervention (ie, fixed bid contract) rather than expenses charged by hours of time worked. Contracts in which expenses are billed from the vendor to the research grant that are for the number of hours worked can quickly balloon and extend beyond the original budget estimate. This was especially a concern for grants that had a large initial budget, where costs may not be tracked closely:

We were under the assumption that we were getting a package deal for this dollar amount. What we came to find out that no, in fact all of the revisions, all of the work that we were having them do, that was adding on hours and our budget was simply no longer. I wish that would have been negotiated better at the get go…in the future I will put together a very specific expectations as to how many revisions will we get, how much money can we spend, how much time can we spend, what are deadlines for things.

Coordinator, Transgender HIV Prevention

In contrast, contracts that were clearly specified resulted in a far smoother development process. Several researchers also recommended including language in the contract for funds to support the intervention after it is launched and open for participants to enroll. Holding funds in reserve for technical support will ensure that technical problems that may arise after enrollment has begun will be able to be addressed by the original development team and in a timely manner. Otherwise, the project may be jeopardized by having to take time to renegotiate with the vendor to provide support and finding additional funds for that support.

#### Document All Decisions and Development Activities

A theme that emerged from our discussions was that all decisions and development activities should be carefully documented on an ongoing basis. There were two primary reasons for documenting decisions and activities. First, documentation provided the framework in which the intervention team and vendor could clarify the precise features and functions that were desired for the intervention. Intervention teams often found it difficult to clarify exactly what features and functions they would like to include in the intervention to the vendor, and creating a document to describe their wishes for features and functions was a starting point to make sure that the intervention features are built as wanted:

Where some of the difficulty came in was that neither the PI nor myself had experience really developing an online intervention in that way and so we didn’t speak the same language as [the vendor] and so we would try to say, “this is what we want” and he would try to create that and it wouldn’t be exactly what we wanted or we needed…As we started to learn the right language and as we started to learn to what to say we really needed, and then we also started making sure that we wrote it all down in a full document.

Coordinator, Adolescent Sexual Health

The second main reason of documenting all decisions and development activities was to assist with negotiating any potential conflict. Much like having a clear and detailed contract, documenting all decisions allowed for the resolution of conflicts that inevitably arise during the development process:

We discovered pretty early on in the development process that we had to write down every single thing that was agreed upon between the web vendor and ourselves…We would say yes, this is what we want. They would say actually that’s going to cost more money. I ended up having to go through hundreds of emails over the course of this study and show documentation of where they had agreed to certain terms and have them follow through on what was promised. So I guess my advice would be to document as much of this process as possible as you’re going through.

Coordinator, Alcohol Policy

In sum, creating a systematic way to document decisions and development activities throughout the entire process is a necessary aspect of the development process, albeit time consuming for research staff. However, this investment is time well spent for preserving and enhancing the functionality of the Web product and staying on budget.

#### Use a Content Management System

A content management system (CMS) is an interface that allows users to edit and publish content from a central portal. Users can be anyone designated to make changes to the content (eg, study coordinator or research assistants). While the CMS is primarily used to edit and modify written content on the website, CMSs may include visual content (eg, video or pictures) or audio clips. The primary advantage of the CMS was that it allowed the research team to easily make changes to the website without requesting changes through the vendor:

The good thing about a content management system is that you can change your content and keep uploading new stuff or work through it. But it took, because the website is so large, and because we have a couple versions of the website, it took at least a month to upload all the content.

Coordinator, Alcohol Policy

The alternative to allowing the research team to make changes to the website content through the CMS was to report changes to the vendor, who would make the edits to the code and subsequently wait for feedback from the intervention team. This process was both time consuming and costly since vendors charged for making such changes. Whichever process is used, allowing sufficient time and staff resources to effectively utilize the CMS was critical to the success of the study.

### Test and Refine

#### Overview

The Test and Refine component of the usability model refers to the part of the process when the intervention is examined for technical issues and refined for final rollout. This process often includes internal testing (ie, testing by members of the intervention and/or vendor, often referred to as “alpha” and “beta” testing) and testing with members of the target population (also known as usability testing) [27-29]. As usability testing options have been described elsewhere [19] and the type of usability testing is unique to each study, we do not explore themes related to this part of the process. However, the following theme emerged with respect to the process of internally testing the intervention to prepare for usability and final launch of the intervention.

#### Allow Extra Time for Testing and Debugging Your Intervention

All researchers interviewed for the purposes of this study noted that the process of internal testing of the intervention to identify technical problems (ie, debugging) took much longer than anticipated. All the researchers noted that even though the contract with the vendor stated that the vendor team would conduct internal testing to identify and correct technical errors, errors and problems with the functionality of webpages and features were common. This was frustrating for many researchers interviewed here, as they did not anticipate having to use resources and time to conduct their own testing:

We should not have been doing beta testing, [the vendor] should have been doing that. But they acknowledged that in the end…we explained that this was really one of the most difficult things on our end to want to be starting the study and to find out that things weren’t working. Even when we were going live, having things that weren’t functioning properly.

PI, Adolescent Sexual Health

Furthermore, the timeline for the process of testing was often extended because it often took longer than anticipated to fix bugs, and occasionally a fix for one technical problem caused another unanticipated bug.

The process of communicating technical problems identified during usability testing varied from study to study. One study used an online interface (similar to a content management system) to communicate “bugs” to the vendor team, as discussed below:

Debugging was basically our research team, coordinator, research assistant and me going in and playing with the website and then just coming up with a list of things that worked and things that didn’t work, and then we’d take it back to [the vendor] and…depending on how much work it was, he would fix it within anywhere from a day to up to a week.

PI, Medication Adherence

Other research teams communicated with the vendor by emailing a list of problems to the vendor and having the vendor indicate on that same list when the problem was fixed:

As I would find bugs, I would create a word document saying what I was finding. Usually you have to include a screenshot of what was wrong, and then I would submit a list every day to the developers. The developers would then add their own answers to each section of the word doc and so they would either say, “yes this has been fixed” or “we can’t fix it because of this, but we made this change instead.

Coordinator, Alcohol Policy

## Discussion

### Principal Findings

The purpose of this study was to provide guidance and lessons learned to researchers and practitioners who are interested in developing online health interventions from the perspective of those who have been involved in such endeavors. Creating a knowledge base of lessons learned in the development of online health interventions is needed since the ultimate success of a particular technology-based intervention rests on attention paid to critical aspects of intervention development prior to launch. The User-Centered Design Process Map [22] was used to guide interviews with principal investigators and project coordinators who had prior experience with online health intervention development. A number of practical tips and suggestions were evident in the findings described here, from which research or other intervention teams would benefit from answering key questions to facilitate the development during various phases of an online intervention (see Table 3). It is also worth noting that the amount of time and effort required for each phase of online intervention development will vary depending on the complexity of the intervention and the extent of evaluation needs for the project. In the discussion below, we draw attention to several overarching points about the process of online health intervention development that appear to be most important from both the interviews that were conducted for this study and our own experience.

**Table 3:** Key questions to consider in phases of the development process for online health interventions.

Phase		Questions
**Plan & Analyze: Intervention conception; team member involvement in the initial planning; hiring the vendor; adaptation of an existing website or curriculum, learning about the target audience, and formative research**		
	Does the study coordinator have experience with online health intervention development and with communicating with vendor?	
	Does the study team include someone with experience in contract negotiations?	
	Does the study team include someone with experience in software programming?	
	Does the research team understand the target population, including their uptake of technologies, their computer literacy, and how they will react to various aspects of the intervention?	
	Did the research team allow enough time to conceptualize goals of the project and develop some of the content prior to meeting with the vendor?	
**Design: Defining the individual components of the websites; discussions about authoring and uploading content; and work processes with the vendor**		
	Has the research team conducted an inventory of their values and objectives for designing the online health intervention?	
	Is there sufficient time to address communication problems that may arise during the development process?	
	Is a detailed contract between the intervention team and the vendor in place prior to beginning development?	
	Does the contract provide specific language about the scope of work?	
	Are there sufficient funds remaining to pay the vendor to maintain the website after launch?	
	Is there a system in place to document all decisions made between the research and vendor teams?	
	Will the intervention have a content management system and is there sufficient time to upload content for the intervention?	
**Test & Refine: Internal testing; usability testing; and de-bugging**		
	Is sufficient time set aside during internal testing and de-bugging?	
	What are the processes for reporting bugs to the vendor and ensuring that bugs are fixed?	

It is critical to consider the values held by members of each participating organization (eg, researchers, government agencies, or vendors) involved in the development of the online intervention or program, as well as the objectives that are trying to be met with the website. The values held by researchers interviewed here are reflected in the themes and quotes described earlier and are exemplified in Table 2. For example, values held by health researchers include advancing understanding of complex behaviors and events, and using the scientific inquiry to improve life expectancy and overall quality of life. The objective of assessing the efficacy of a specific intervention approach reflects these values, which often requires that researchers are entrusted as stewards of federal research grant money and must be responsible to the funding agency and the public. In contrast, for-profit businesses and organizations may value profits and have responsibilities toward employees, owners, or shareholders. For such organizations, their objectives of building a website may be to attract customers, increase visibility of the company and increase sales. The values and objectives of governmental agencies or community-based organizations also may differ in ways from researchers or for-profit companies, and these have implications for how the online health intervention is designed, as shown in the last column of Table 2. For example, since protecting participant identity is critical in research, the importance of blocking access to the online intervention to the public and developing strong security features must be clearly described to the vendor. In comparison, for-profit businesses may wish to widely market their website and encourage browsing of the website by consumers. For these reasons, we recommend that each organization take an inventory of their own values, objectives, and needs as a first step in developing an online health intervention.

With values and objectives of an organization clearly defined, accurately articulating them to the vendor is important so that an appropriate scope of work can be developed and the website can be developed within the allotted budget. All PIs and coordinators interviewed for the purpose of this study highlighted difficulties in communication with vendors as a barrier to developing the online intervention that was initially intended or conceived by the research team and/or staying within the initial timeline or budget. Common areas of miscommunication included whether a component of the intervention had been fully tested and who was responsible for testing, whether the vendor charged for their time by the hour or by the entire project, and how quickly (ie, the number of days) the intervention team should provide feedback to the vendor about a developed intervention component to ensure that the timeline is met. Thus, we recommend open and explicit discussion about these and other needs from the perspectives of the intervention team (or whichever organization is driving development of the website) and the vendor.

To the degree possible, details of these discussions should be included in the initial contract between the organization and the vendor to avoid confusion and frustration. Such agreements may also require that each team step outside of its comfort zone. Intervention teams, who may be familiar with taking extended amounts of time to consider different aspects of an in-person intervention, may be asked to make decisions more quickly when working with a commercial vendor to develop online interventions. Moreover, intervention teams may be comfortable altering different features of an in-person intervention well into the development of the intervention. In contrast, altering features or functions of online interventions after they have been programmed and completed is both costly (since additional programming will need to be paid for) and time consuming.

The points noted above highlight the importance of researchers and practitioners becoming familiar with online intervention development, and how it may differ from the development and implementation of in-person interventions or programs. To learn the nuances of Web-based intervention or program development, the intervention team should consider expanding the team to include experts in computer science or learning technologies, including outside consultants with skills and expertise who can manage specific aspects of the study (eg, a consultant with expertise in contract development and negotiations), and taking advantage of institutional resources that will be needed for successful completion of the project. Overall, careful consideration should be given to the composition of the team and should include members who have expertise outside of traditional health fields to successfully oversee the project. Once the appropriate team and resources are identified, this information may be overlaid with the timeline to introduce these team members and resources at the appropriate time.

Finally, we wish to draw attention to the tradeoff between funds available for online intervention or program development and the complexity of the project. We interviewed researchers and coordinators who participated in studies with a range of budget caps, from those with relatively small budgets (eg, US $40,000) to those with much larger budgets (eg, US $200,000 or more). It was evident that the greater availability of funds translates into websites that are more customizable and dynamic in appearance and functionality. All of the vendors hired by research teams with more than US $200,000 of funds available for development were able to hire a vendor with a large team of persons, each with unique skill sets. As such, interventions that had relatively large budgets available for development resulted in interventions that were complex and dynamic compared to those with relatively small budgets. However, because of their complexity, research studies with large development budgets were also at greater risk for going beyond their initial budget estimate and for miscalculating the timeline. These tradeoffs between affordability, complexity, and adherence to budget and timeline will need to be considered by all stakeholders, including funding agencies, researchers/practitioners, and vendors.

As Internet use becomes more ingrained in people’s lives, users are becoming more technologically savvy and altering how they search for and use technology [30,31]. As such, end users of online health interventions may have increasing expectations that such programs be tailored, engaging, and sophisticated. Several persons we interviewed for this study noted that the final online intervention did not meet the needs and expectations of their target population, resulting in unexpectedly low engagement with the intervention. As such, we and others [2] recommend consistent consideration of features and functions that will maintain continual website utilization over time among its end users. This may be done by seeking input from the end user to assess their technology expectations and use prior to beginning development, as well as obtaining feedback about the look and feel of the intervention or program throughout the development period. Above all, planning for adequate budgets and development of products that incorporate expected features is important from the outset of a project.

### Limitations

This study has several limitations that affect the generalizability of the findings. First, we interviewed a small number of researchers involved in the development of online health interventions, including authors of this study. We did not intend for the results to represent the experiences of all persons involved in online health intervention or program development. Rather, the purpose of this study was to share our own and others’ experiences with developing Internet-based health interventions to provide possible guidance to other stakeholders interested in developing their own online health intervention or program. We believe that this study represents the collective experience of researchers across a variety of different health topics, and the themes and recommendations highlighted here will provide important cautionary considerations in the development of online health interventions and programs that are not typically found in current literature. Second, we interviewed only researchers involved with online health interventions at one institution. Greater or fewer resources may be available at other institutions, agencies, or organizations to develop online health interventions, and some themes and recommendations noted here may not be relevant for persons in other settings. We encourage readers to assess their own institutional capacity and resources to determine whether the recommendations made here are relevant. Third, we did not interview vendors to gain their perspective about the process of developing online health interventions. We encourage future researchers to interview vendors and compare their experiences to those described by research teams in this study to contribute to a fuller understanding of the development process.

### Conclusions

The results of this study serve as important reminders of the complexity of developing online health interventions. Many of the procedures and practices commonly used to develop traditional, in-person interventions do not translate seamlessly to the development of Internet-based intervention development. We believe that the themes and recommendations put forth in this study will assist researchers and practitioners to more successfully navigate the complex process of online health intervention development.

## Abbreviations

CMS: content management system
HIV: human immunodeficiency virus
MSM: men who have sex with men
PI: principal investigator
